# Highly Sensitive Gas Sensing Material for Environmentally Toxic Gases Based on Janus NbSeTe Monolayer

**DOI:** 10.3390/nano10122554

**Published:** 2020-12-19

**Authors:** Deobrat Singh, Rajeev Ahuja

**Affiliations:** 1 Condensed Matter Theory Group, Materials Theory Division, Department of Physics and Astronomy, Uppsala University, Box 516, 75120 Uppsala, Sweden; deobrat.singh@physics.uu.se; 2 Applied Materials Physics, Department of Materials Science and Engineering, Royal Institute of Technology (KTH), S-100 44 Stockholm, Sweden

**Keywords:** electronic properties and charge transfer, first-principles calculations, gas sensors, *I* − *V* characteristics, Janus NbSeTe monolayer, sensitivity

## Abstract

Recently, a new family of the Janus NbSeTe monolayer has exciting development prospects for two-dimensional (2D) asymmetric layered materials that demonstrate outstanding properties for high-performance nanoelectronics and optoelectronics applications. Motivated by the fascinating properties of the Janus monolayer, we have studied the gas sensing properties of the Janus NbSeTe monolayer for CO, CO${}_{2}$, NO, NO${}_{2}$, H${}_{2}$S, and SO${}_{2}$ gas molecules using first-principles calculations that will have eminent application in the field of personal security, protection of the environment, and various other industries. We have calculated the adsorption energies and sensing height from the Janus NbSeTe monolayer surface to the gas molecules to detect the binding strength for these considered toxic gases. In addition, considerable charge transfer between Janus monolayer and gas molecules were calculated to confirm the detection of toxic gases. Due to the presence of asymmetric structures of the Janus NbSeTe monolayer, the projected density of states, charge transfer, binding strength, and transport properties displayed distinct behavior when these toxic gases absorbed at Se- and Te-sites of the Janus monolayer. Based on the ultra-low recovery time in the order of μs for NO and NO${}_{2}$ and ps for CO, CO${}_{2}$, H${}_{2}$S, and SO${}_{2}$ gas molecules in the visible region at room temperature suggest that the Janus monolayer as a better candidate for reusable sensors for gas sensing materials. From the transport properties, it can be observed that there is a significant variation of I−V characteristics and sensitivity of the Janus NbSeTe monolayer before and after adsorbing gas molecules demonstrates the feasibility of NbSeTe material that makes it an ideal material for a high-sensitivity gas sensor.

## 1. Introduction

The continuous increase in toxic air pollutants, which has main sources such as emissions from coal-fired power plants, industries, refineries, building materials like asbestos, tobacco smoke, and chemicals like solvents as well as from transportation, is the biggest problem in today’s era [1,2,3,4]. Gases and vapors produced under any circumstances have harmful effects on workers exposed to them when inhaled, absorbed through the skin, or ingested [5,6,7]. Therefore, the detection of these toxic gases has become immensely essential for environmental monitoring, our own safety, and manufacturing/industrial monitoring products [8]. It was seen that the traditional gas sensors based on semiconductor oxide materials displayed better performance in terms of sensitivity while it operates at high temperature, i.e., 200–600${}^{\circ }$C which creates several problems, for example high power consumption and other safety-related problems [9]. These restrictions have led researchers to produce highly sensitive gas sensors that can operate at room temperature. For this regard, 2D layered materials of graphene [10,11], other monoelemental materials such as phosphorene [12], silicene [13], germanene [14], antimonene [15], indiene [16], arsenene [17], etc., transition metal dichalcogenides (TMDs) [18,19,20], MXenes, and other layered materials [21,22,23] have received significant attention. These predicted layered materials displayed extraordinary electrical, optical, photocatalysts, thermoelectric, and magnetic properties at single as well as multi-layer levels which have been integrated into gas detection devices.

2D materials are supposed to be promising candidates for chemical sensing materials because of their large surface/volume ratio and performance at room temperature [24]. The basic mechanism for gas detection can be understood in different ways: variation of resistance, significant changes in work functions, and vibrational frequencies [25,26]. The basic mechanism of resistance variation by the adsorption of gas molecules is mainly associated with electron charge transfer between the gas molecules and 2D layered materials. The principle mechanism of work function variation of 2D materials is produced by the adsorption of gas molecules on 2D layered materials. Additionally, the underlying principle of vibrational frequencies is also the criteria for gas detection which is associated with the favorable chemical bonding interactions of gas molecules on the 2D layered substrate induce significant changes of vibrational properties of the bare gases. Apart from this, researchers developed a different way to change the transport characteristics of 2D materials, for example mechanical strain, light, and gate voltage, to improve the performance of gas detection of 2D materials [27,28,29]. The previous investigations have suggested that the effect of these modulations is very limited [30,31]. The new family of 2D materials named Janus transition metal chalcogenides (TMDs) monolayer has been recently synthesized [32,33]. Due to the asymmetry sandwich combinations (i.e., X-M-Y, where M = transition element, X, Y = chalcogen atoms but X ≠ Y) of Janus TMDs monolayer have very interesting physical and chemical properties [34,35,36,37,38,39]. Additionally, due to the quite different atomic radius and electronegativities of X and Y atoms, the charge distributions of the layer X-M and M-Y in Janus monolayer are significantly different [34,35,40]. As a consequence, Janus MXY monolayer generates interior electric fields transverse to the surface [41]. The Janus monolayer has an interior electric field similar to the external electric field built up by gate bias [42]. Generally, sensing devices are mainly operated by additional gate bias. From this point of view, Janus monolayer has excellent potential candidates to realize superior sensitivity without gate bias. Some of the work has been reported on various gas molecules detection for gas sensing devices [35,43,44,45,46,47,48,49]. Still, there is a need for a better understanding of various gas sensing properties of the Janus monolayer.

Motivated by this, we explore the interaction between the Janus NbSeTe monolayer and adsorbate gas molecules to better understand the capabilities of the Janus-based nanosensor devices. In this manuscript, we report the first-principles density functional theory (DFT) calculations for considered toxic gases, i.e., CO, CO${}_{2}$, NO, NO${}_{2}$, H${}_{2}$S, and SO${}_{2}$ molecules on Janus NbSeTe monolayer. We have investigated the different positions with the possible orientation of these gases to check the favorable binding energies. The strength of binding energies strongly depends on the significant amount of charge transfer and variation of work function with the adsorption of gas molecules on the Janus surface. Similar to 2D layered materials, our finding results displayed the better binding strength of gas on Janus NbSeTe monolayer with a noticeable amount of charge transfer which indicates superior sensitivity. Furthermore, for gas sensing device modeling, we have investigated the current–voltage (I−V) characteristics for the gas molecules on the Janus substrate with the help of the non-equilibrium Green function formalism (NEGF) method. The significant variation in the I−V profile suggests that the Janus-based nanosensor device is a better candidate for distinct ON/OFF states.

## 2. Computational Methods

The electronic structure calculations have been performed using first-principles calculations as implemented in the Vienna Ab-initio Simulation Package (VASP) software [50,51,52]. The generalized gradient approximation (GGA) in the form of Perdew–Burke–Ernzerhof functional (GGA-PBE) has been used for an exchange-correlation interaction [53]. The long-range van der Waals density functional (vdW-DF) were considered by Dion et al. [54]. For the plane-wave basis set, we have used an energy cutoff of 600 eV and (21 × 21 × 1) k-meshes for (1 × 1 × 1) unit cell of NbSeTe monolayer system for Brillouin zone integration within the Monkhorst–Pack scheme [55]. To describe the ion–electron interaction, we have used the Projected augmented wave (PAW) potential [56]. Furthermore, we have used the (4 × 4 × 1) supercell with (6 × 6 × 1) k-meshes for the adsorptions of toxic gases on both sides of the NbSeTe monolayer. In addition, a vacuum of 20 Å has been used in the perpendicular directions to prevent the physical interactions between the periodic images. The convergence criteria for Hellmann–Feynman force [57] fell below 5 × 10${}^{-3}$ eV/Å during the structural optimizations. The energy convergence criterion for the electronic wave function has been set as 10${}^{-6}$ eV.

To further investigate the potential applications of the Janus NbSeTe monolayer for the sensing of toxic gases, the electronic transport properties have been performed before and after toxic gas adsorption on the surface of the Janus NbSeTe monolayer based on non-equilibrium Green function (NEGF) using Transiesta module of the Siesta code [58,59]. In the Siesta code, 400 Ry energy cutoff and double-ξ polarized (DZP) basic set with vdW-DF by Dion et al. [60] has been used for toxic gas sensing absorbed on the surface of the Janus NbSeTe monolayer. For the geometry, optimization is achieved when the maximum difference between the output and input of each item of the density matrix is less than $10^{-4}$. During the electronic transport properties calculations, we have used Monkhorst–Pack k-point grids of (10 × 1 × 100) for electrode and 10 × 1 × 1 for full device modeling. To demonstrate the probability that electrons are transferred to the electrode from left to right with definite energy *E* for transmission spectrum is as follows [61],

(1) $$T\left(E,V\right)=tr[\Gamma_{R}\left(E,V\right)G^{R}\left(E,V\right)\Gamma_{L}\left(E,V\right)G^{A}\left(E,V\right)],$$

Here, Γ and $G^{A}$ represents the coupling matrix of electrodes on either side and Green’s function of the central region. Integration of this transmission function gives the electric current through the device atomic scale, calculated from formula of Landauer–Buttiker,

(2) $$I\left(V_{b}\right)=G_{0}\int_{\mu_{L}}^{\mu_{R}}T\left(E,V_{b}\right)\left[f\left(E-\mu_{L}\right)-f\left(E-\mu_{R}\right)\right]dE,$$

where $V_{b}$, $\mu_{L}$ and $\mu_{R}$ represents the applied bias voltage, electrochemical potentials of left and right electrodes, respectively. Moreover, $T(E,V_{b})$ displayed the transmission coefficient at energy *E* and applied bias voltage $V_{b}$, f(E) represents the Fermi–Dirac distribution function, and $G_{0}$(=$2e^{2}/h$) shows the quantum conductance. The above Equations (1) and (2) are used to calculate the transmission spectra and current–voltage (I−V) characteristics of device modeling for toxic gas sensing on the surface of the Janus NbSeTe monolayer.

## 3. Results and Discussion

### 3.1. Structural and Electronic Properties

**Figure 1:**
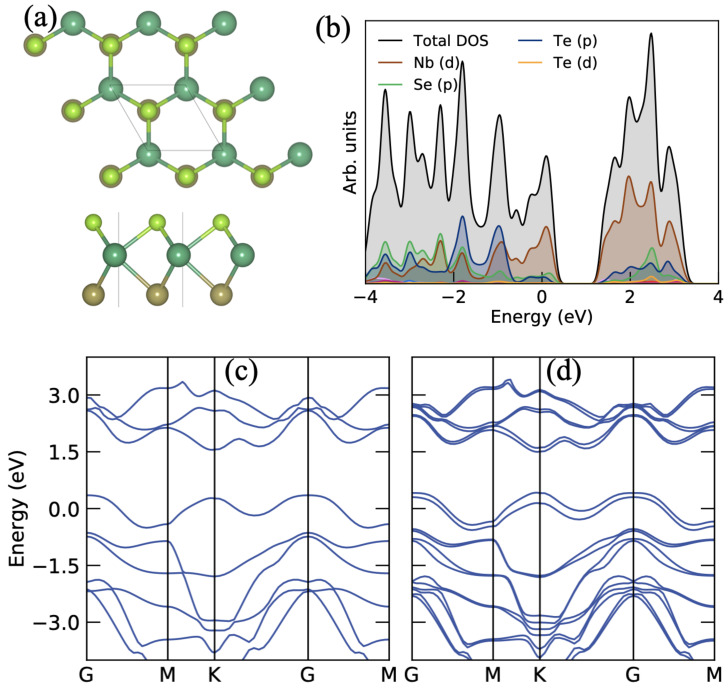
(**a**) Optimized structure with top and side view, (**b**) projected density of states, (**c**) electronic band structure without spin-orbit coupling, and (**d**) electronic band structure with spin-orbit coupling of the Janus NbSeTe monolayer.

The optimized structure with top and side view of the Janus NbSeTe monolayer is shown in Figure 1a. The hexagonal arrangement of atoms in the unit cell contains three atoms (single atom of Nb, Se, and Te). The optimized lattice parameter is a = b = 3.58 Å, corresponding bond lengths between Nb-Se and Nb-Te are 2.61 Å and 2.82 Å, respectively and bond angle between Se-Nb-Te is 80.32${}^{\circ }$ which is consistent with previous reported work [35,62,63]. In addition, it was seen that the lattice constant of the Janus NbSeTe monolayer lies between the TMDs NbSe${}_{2}$ (a = b = 3.48 Å) and NbTe${}_{2}$ (3.70 Å) monolayer [63]. The thickness of the Janus NbSeTe monolayer of 3.51 Å. Moreover, our previous work on Janus NbSeTe monolayer displayed that it is energetically and dynamically stable [35]. Furthermore, to see the electronic properties, we have calculated the projected density of states (PDOS) and electronic band structure as shown in Figure 1b–d. From the PDOS, we can see that the Nb-d orbitals mainly contribute to the Fermi level and a very less contribution comes from the p-orbitals of Se and Te atoms. In addition, the bottom of the conduction band is made by Ti-d orbitals (see Figure 1b). In addition, from the electronic band structure, the single band line crosses the Γ and K points at the Fermi level as shown in Figure 1c. Due to that, the Janus NbSeTe monolayer shows metallic behavior. Figure 1c,d shows the electronic band structures without and with spin-orbit coupling (SOC). With the inclusion of a relativistic effect, the electronic bands significantly split (see Figure 1d).

### 3.2. Binding of Toxic Gases on NbSeTe Monolayer

**Figure 2:**
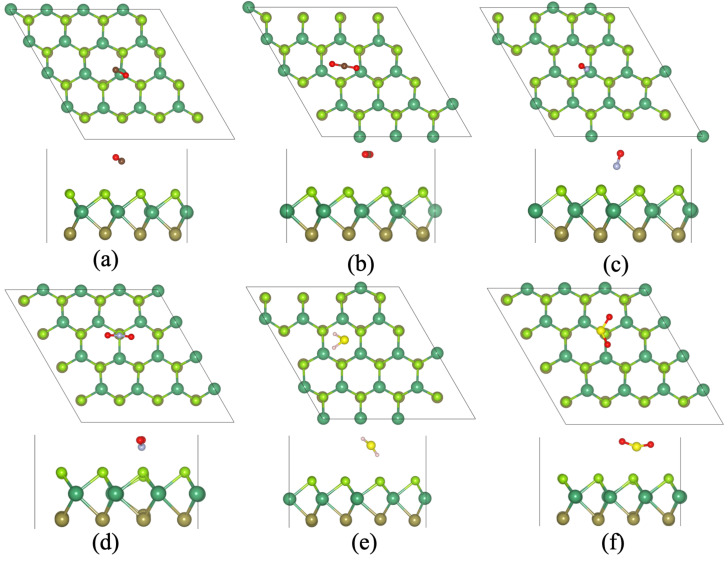
Optimized structure of toxic gas molecules adsorbed on the surface of the Janus NbSeTe monolayer with the top and side view. (**a**) CO, (**b**) CO${}_{2}$, (**c**) NO, (**d**) NO${}_{2}$, (**e**) H${}_{2}$S, and (**f**) SO${}_{2}$ gas molecules adsorbed on Se-side of the Janus NbSeTe monolayer.

**Figure 3:**
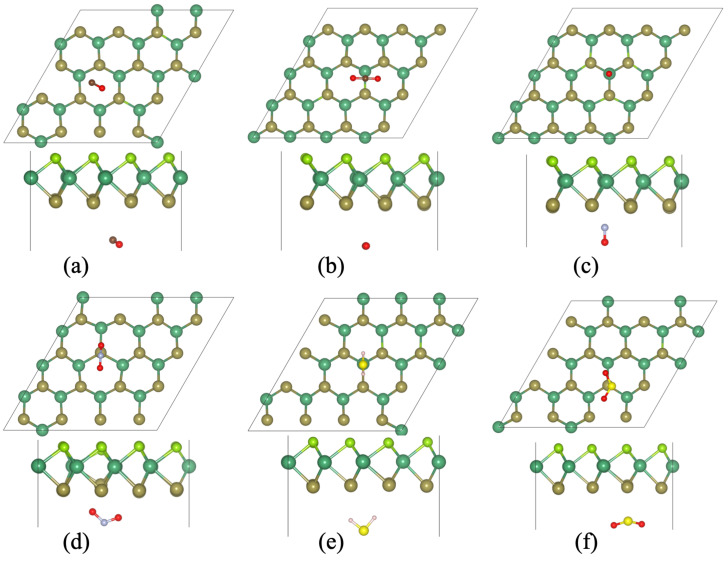
Optimized structure of toxic gas molecules adsorbed on the surface of the Janus NbSeTe monolayer with the top and side view. (**a**) CO, (**b**) CO${}_{2}$, (**c**) NO, (**d**) NO${}_{2}$, (**e**) H${}_{2}$S, and (**f**) SO${}_{2}$ gas molecules adsorbed on the Te-side of the Janus NbSeTe monolayer.

First, we have checked the lowest adsorption sites of these toxic gas molecules on the surface of the Janus NbSeTe monolayer. Due to the asymmetric nature of the Janus NbSeTe monolayer, we have considered these gas molecules on both sides because the top and bottom side has Se and Te atoms. Initially, we have check eight possible adsorption sites in which the most favorable configurations are presented in Figure 2 and Figure 3. To better understand the behavior of sensing characteristics, we have calculated the binding height, adsorption energies, and charge transfer between Janus NbSeTe monolayer and toxic gases, i.e., CO, CO${}_{2}$, NO, NO${}_{2}$, H${}_{2}$S, and SO${}_{2}$ molecules. The binding energy/adsorption energy of toxic gas molecules with Janus NbSeTe monolayer was calculated by the following relation,

(3) $$E_{b}=E_{\left(\mathrm{NbSeTe}+\mathrm{gas}\right)}-E_{\mathrm{NbSeTe}}-E_{\mathrm{gas}},$$

where $E_{\left(\mathrm{NbSeTe}+\mathrm{gas}\right)}$, $E_{\mathrm{NbSeTe}}$, and $E_{\mathrm{gas}}$ represent the total energy of the Janus NbSeTe monolayer with gas molecule, Janus NbSeTe monolayer, and isolated gas molecules. According to Equation (3), the negative value of the binding energy means that the adsorption process is energetically favorable and the molecular gas is strongly bound to the monolayer.

For the adsorptions behavior of these toxic gas molecules, we have considered a (4 × 4 × 1) supercell with the surface areas of 14.26 Å × 14.26 Å. It is clear that these toxic gas molecules display a similar preference for the adsorption sites and the configuration of adsorption on the same side. It was seen that the adsorption energies of these gas molecules CO, CO${}_{2}$, NO, NO${}_{2}$, H${}_{2}$S, and SO${}_{2}$ displayed negative values, which means that each of the gas molecules are energetically favorable on the surface of the Janus NbSeTe monolayer. As previously reported by Hussain et al., the critical range of binding energies should be −0.30 eV to −0.80 eV, to achieve an appropriate amount of gas adsorption on any given surface of the material [64]. In our case, the range of binding energies lies between −0.12 eV to −0.68 eV as shown in Table 1. The adsorption energies of these toxic gas molecules on the Se-side are slightly larger than that of the Te-side for surface sensitivity of the Janus NbSeTe monolayer (see Table 1). Additionally, the adsorption energies of NO and NO${}_{2}$ gas molecules have the lowest energy configurations on both side (Se and Te-side) as compared to CO, CO${}_{2}$, H${}_{2}$S, and SO${}_{2}$, due to the more electronegativity of N and O atoms as shown in Table 1, Figure 2 and Figure 3. Based on these values, Janus NbSeTe monolayer is expected to show more affinity towards NO and NO${}_{2}$ gas molecules with the highest binding strength.

Initially, we have optimized the isolated toxic gas molecules to determine the interaction behavior between gas molecules, i.e., CO, CO${}_{2}$, NO, NO${}_{2}$, H${}_{2}$S, and SO${}_{2}$ and Janus NbSeTe monolayer for that these gases were placed at a vertical distance of 2 Å from the Janus surface. The bond lengths between C–O, N–O, H–S, and S–O of these isolated gas molecules are 1.14, 1.17, 1.14, 1.21, 1.33, and 1.43 Å for CO, CO${}_{2}$, NO, NO${}_{2}$, H${}_{2}$S, and SO${}_{2}$, respectively. In addition, the bond angles in triatomic gases such as CO${}_{2}$, NO${}_{2}$, H${}_{2}$S, and SO${}_{2}$ are 180.0, 133.7, 92.1, and 119${}^{\circ }$, respectively. When these gas molecules adsorbed on the surface of the Janus NbSeTe monolayer, the physical and chemical interactions of these gas molecules with the surface significantly influence the bond length, bond angle in triatomic gases, and vertical distance between Janus surface and gas molecules. In the case of CO gas molecule, the bond length between C and O atoms changes to 1.422 Å which is slightly influenced on both the Se- and Te-side of the Janus NbSeTe monolayer. After the interaction of the CO molecule with the Janus surface, the CO molecule shifted vertically at 2.89 for Se-side and 3.05 Å for Te-side from 2 Å. During the structural optimization, the C atom in the CO molecule is oriented towards the Se- and Te-side of the Janus NbSeTe monolayer as presented in Figure 2a and Figure 3a. Due to the almost similar Pauling scale strength, the adsorption energy is very small with the values of −0.15 eV and −0.12 eV for Se- and Te-side, respectively which is relatively larger than that of previously reported work on graphene [65,66]. From the Bader charge analysis, the CO gas molecules gain 0.0159 and 0.0065 e${}^{-}$ on the Se- and Te-side of the Janus NbSeTe monolayer, respectively.

It was observed that the CO${}_{2}$ molecule prefer parallel orientation to the Janus surface on both side (see Figure 2b and Figure 3b). The optimized structure with the absorption of CO${}_{2}$ molecule slightly changes the bond length of 1.76 Å and the angle between O–C–O is 179.63 and 179.60${}^{\circ }$ for Se- and Te-side, respectively. When CO${}_{2}$ molecule interacts with Janus NbSeTe monolayer then it shifts 3.11 and 3.27 Å in the perpendicular direction for Se- and Te-side, respectively. Similar to the CO molecule, it has a lower binding energy of −0.20 eV and −0.19 eV on the Se- and Te-side of the Janus surface, respectively. The calculated binding energy is higher than that of previously reported work on graphene, silicene, germanene [65,66,67,68]. In addition, it shows 0.0157 and 0.0075 e${}^{-}$ gain from the Se- and Te-side of the Janus surface to CO${}_{2}$ molecule, respectively. Similarly, when NO and NO${}_{2}$ gas molecules are adsorbed on the surface of the Janus NbSeTe monolayer then they will significantly change the bond length and angles between the atoms. The corresponding bond length for Se- and Te-side is 1.16 and 1.17 Å for NO molecule, respectively. During the optimization of NO gas molecule, the N atom orients towards the Janus NbSeTe surface side and the corresponding vertical height shifts towards the Janus surface with the values of 1.95 and 1.81 Å for Se- and Te-side, respectively. It was also seen that the maximum charge transfer was found from the Janus surface to the NO gas molecule because N atoms have more Pauling scale strength. The binding energy and charge transfer are found to be −0.55 eV (−0.53 eV) and 0.1297 e${}^{-}$ (0.024 e${}^{-}$) for Se(Te)-side, respectively. It was seen that the calculated binding energy is better as compared to previous reported values on layered system [14,65,66,67,68]. While the NO${}_{2}$ gas molecule adsorbed on the surface of the Janus monolayer, we found the binding strengths of −0.59 and −0.68 eV on the Se- and Te-side which are relatively better sensitivity as compared to previously reported values on graphene, phosphorene, germanene, and MoS${}_{2}$ [14,65,66,69,70,71]. From these values, we can see that the adsorption energy on the Te-side is larger than the Se-side because the Pauling scale strength of O atom is higher than that of N atom in the optimized NO${}_{2}$ gas molecules oriented towards the Te-side. The relaxed structure displayed the bond length of 1.22 Å on the Se-side while 1.22–1.29 Å on the Te-side and corresponding angles of 129.40 and 117.98${}^{\circ }$ on the Se- and Te-side of the surface of the Janus monolayer, respectively. In addition, the gas molecule shifted near to 1.96 and 1.88 Å in the case of Se- and Te-side, respectively. From these values, Janus NbSeTe monolayer is strongly affected by the NO${}_{2}$ gas molecule on both sides. Due to the strong interaction between the NO${}_{2}$ molecule and Janus NbSeTe monolayer, the Janus surface transfers 0.284 and 0.533 e${}^{-}$ to the gas molecule on the Se- and Te-side, which is relatively higher than the other considered system (see Figure 2d and Figure 3d). Apart from this, the H${}_{2}$S and SO${}_{2}$ gas molecules have lower binding strength as compared to N-containing gases (see Table 1). The absorbed H${}_{2}$S and SO${}_{2}$ gases shifted upwards from the Janus monolayer as presented in Figure 2e,f and Figure 3e,f, respectively. The corresponding bond lengths and angles are 1.355–1.359 (1.466–1.467) Å and 91.03–91.46 (118.08–118.27)${}^{\circ }$ for H${}_{2}$S and SO${}_{2}$ molecules for the Se- and Te-side of the Janus NbSeTe monolayer, respectively. Due to the similar Pauling scale strength, it has binding strength of −0.26 and −0.25 eV and corresponding charge transfer of 0.1614 and 0.135 e${}^{-}$ from the Se- and Te-side on Janus surface to gas molecules, respectively.

**Figure 4:**
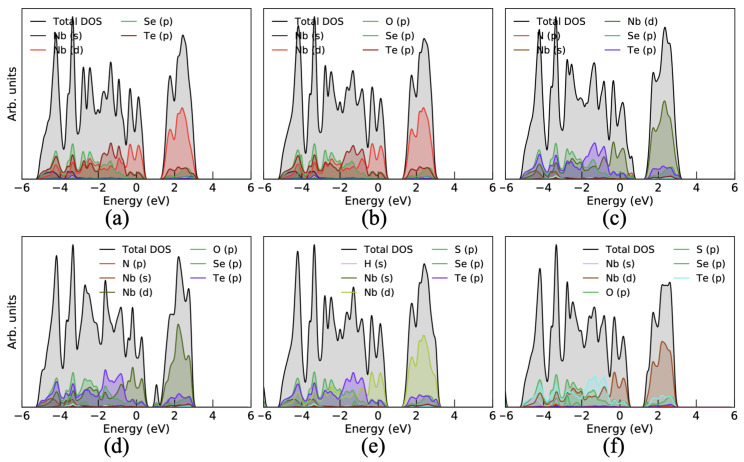
Projected density of states (PDOS) influenced by the toxic gas molecules adsorbed on the surface of the Janus NbSeTe monolayer with top and side view. (**a**) CO, (**b**) CO${}_{2}$, (**c**) NO, (**d**) NO${}_{2}$, (**e**) H${}_{2}$S, and (**f**) SO${}_{2}$ gas molecules adsorbed on the Se-side of the Janus NbSeTe monolayer.

**Figure 5:**
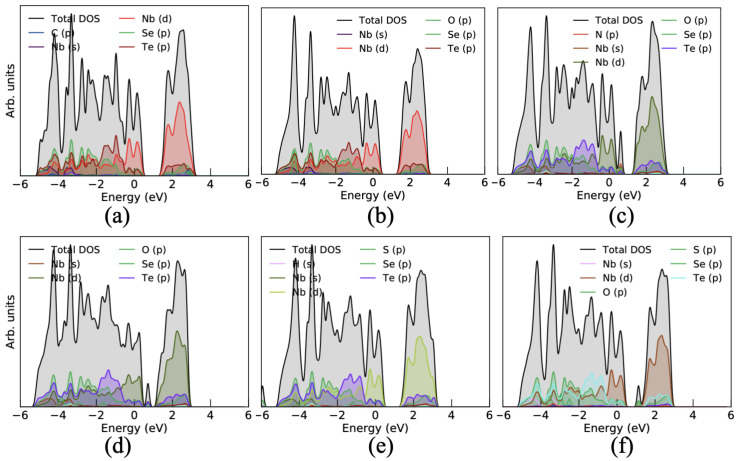
Projected density of states (PDOS) influenced by the toxic gas molecules adsorbed on the surface of the Janus NbSeTe monolayer with top and side view. (**a**) CO, (**b**) CO${}_{2}$, (**c**) NO, (**d**) NO${}_{2}$, (**e**) H${}_{2}$S, and (**f**) SO${}_{2}$ gas molecules adsorbed on the Te-side of the Janus NbSeTe monolayer.

Furthermore, to better understand the influence of these gas molecules on the surface of the Janus monolayer, we have calculated the projected density of states (PDOS). It was seen that the CO gas molecules interact with the Janus surface and slightly change the electronic states of Nb-d states and Se/Te-p states for gas molecule adsorbed on the Se- and Te-side (see Figure 4a and Figure 5a). Due to the small binding energy and less charge transfer between the CO gas molecule and the Janus surface, it shows a small variation in PDOS. In the case of CO${}_{2}$, the gas molecule adsorbs on the Janus monolayer then only p-states of O atom are contributed at the Fermi level as well as above the Fermi level around 2 eV for both Se/Te-side as shown in Figure 4b and Figure 5b. The O atom in CO${}_{2}$ gas molecule is mainly contributed because the O atom is responsible for charge sharing between the Janus surface and the gas molecule. In addition, when the NO molecule interacts with the Se/Te-sides of the Janus surface, then it strongly hybridized with NO gas molecules and some extra electronic states above the Fermi level around 1 eV appears. It also enhanced the electronic states in the valence band near −1 eV as presented in Figure 4c and Figure 5c, respectively, which reflect the larger binding energy and more charge transfer between them. Apart from this, the NO${}_{2}$ molecule also influenced the electronic states and strongly hybridized p-states of O and N with Janus electronic states which appear at 1 eV in the conduction band for both sides of the Janus surface. The electronic states significantly modulated due to the larger binding energy and charge transfer between the Janus NbSeTe substrate and gas molecules (see Figure 4d and Figure 5d). However, for other gases, H${}_{2}$S and SO${}_{2}$, we can see that these molecules induce changes in the DOS (broaden the peak) particularly around the Fermi level, which can be easily inferred by comparing the pure Janus NbSeTe monolayer DOS as depicted in Figure 4e,f and Figure 5e,f for the Se/Te-side, respectively.

Additionally, we have calculated the work function (ϕ) of the pure Janus monolayer as a reference and Janus monolayer with gas molecules, which is an important quantity in the field of gas detection applications. The work function can be defined as the minimum energy required to remove an electron from the Fermi level to infinity which is defined by [64,72],

(4) $$\phi =V_{\infty }-E_{f},$$

where $V_{\infty }$ represents the electrostatic potential at infinity and $E_{f}$ is the Fermi level. The value of ϕ of the pure Janus NbSeTe monolayer is found to be 5.15 eV, which changes significantly when it comes in contact with these toxic gas molecules. It has been reported that the conductivity of materials, especially the 2D layered system, is directly related to the change in the work function. A comparative analysis of ϕ value calculated for different systems clearly shows a variation that might be combined with the change in conductivity after exposure of these toxic gas molecules CO, CO${}_{2}$, NO, NO${}_{2}$, H${}_{2}$S, and SO${}_{2}$ as presented in Table 2.

Additionally, the recovery time τ for sensitivity and selectivity is an important quantity to design an efficient nanosensor. Relatively smaller values of τ demonstrate the reversibility of the gas sensor. Here, we discuss briefly the values of τ for Janus monolayer with gas molecules, which determines its reversibility and could be calculated by the theory of the transition state as given by,

(5) $$\tau =\omega^{-1}exp\left(-E_{b}/kT\right),$$

where ω is the attempted frequency, $E_{b}$ is the binding energy/adsorption energy, *k* and *T* represents the Boltzmann’s constant and operational temperature, respectively. A small value of adsorption energy of toxic gas molecules on the Janus substrate significantly reduces the recovery time and is responsible for a better reversible sensor. In this case, we have taken the value of ω is 10${}^{14}$ $s^{-1}$ in visible region which is reported in various literature [73]. When the values of $E_{b}$ in Equation (5) are used for CO, CO${}_{2}$, NO, NO${}_{2}$, H${}_{2}$S, and SO${}_{2}$ gas molecules on the Janus monolayer, the corresponding values of τ are presented in Table 2. The calculated recovery time for the considered gases are very short as compared to previously reported literature [64,74,75]. The relatively low value of the recovery time τ strongly suggests that the Janus NbSeTe monolayer may be a good candidate for reusable sensors.

### 3.3. Transport Properties

**Figure 6:**
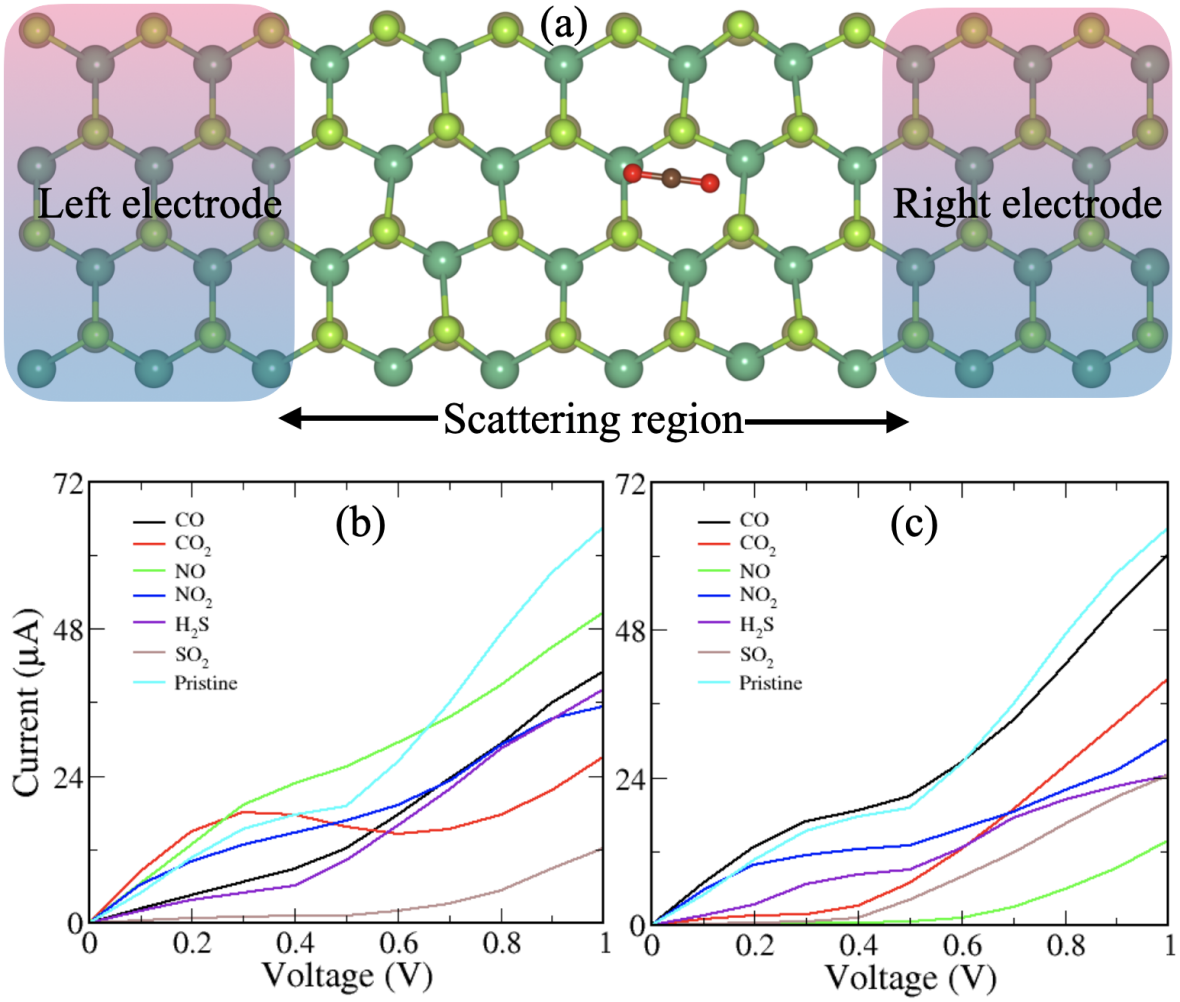
(**a**) Schematic representation of the device model constituted by three parts: the semi-infinite left (L) and right (R) electrodes and scattering region. Current–voltage characteristics of the Janus NbSeTe monolayer for (**b**) gas molecules absorbed on the Se-side and (**c**) gas molecules absorbed on the Te-side.

**Figure 7:**
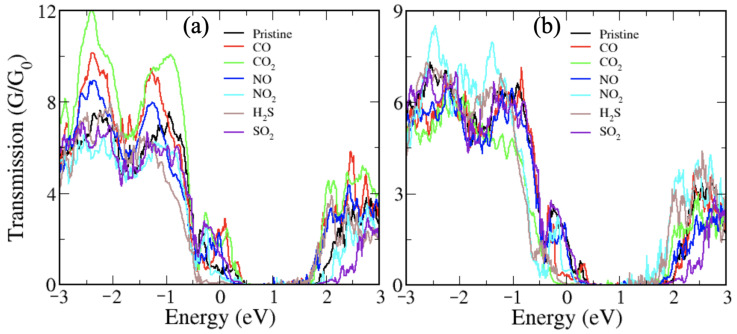
Transmission spectra of the Janus NbSeTe monolayer for (**a**) gas molecules absorbed on the Se-side and (**b**) gas molecules absorbed on the Te-side.

To assess the actual involvement of the material for the toxic gases based on the Janus monolayer as a sensor, it should be explored as a field-effect transistor (FET), wherein varying the primary factor responsive to the voltage or threshold resistance. Therefore, we calculate the transport properties for a wide range of bias (voltage) in order to validate the output of the Janus monolayer with and without gas molecules using the method of the NEGF. We modeled both left and right with the same electrode material and the dispersion region positioned between the two electrodes. The electrode is considered semi-infinite and gas molecules adsorbed are maintained in the scattering region. Electrode/scattering regions are constructed by the Janus monolayer with a two-probe system as presented in Figure 6. Transmission spectra calculated under zero bias gate voltage are shown in Figure 7. It is obvious that there is a region of zero transmission spectra above the Fermi level with a width of ∼1.0 eV for Janus NbSeTe monolayer. Furthermore, this gap is slightly reduced toward the Fermi energy, depending on the different types of adsorbed gas molecules. In addition, it was seen that on the Janus NbSeTe monolayer with adsorbed gas molecules significantly appears distinct peaks around the Fermi level in the energy range of −0.5 eV to 0.5 eV in the transmission spectra. It is produced by the channel conductance available from the various energy bands, which is confirmed by the investigation of the density of states as shown in Figure 4 and Figure 5. Here, some of the conductance channels of the Janus NbSeTe monolayer are partially inhabited with the adsorption of molecules. Correspondingly, the passing current decrease is due to reduced channel conductance.

To check the significant changes in the resistance of the Janus NbSeTe monolayer before and after gas adsorbed molecules, we have simulated the current–voltage (I−V) characteristics. The I−V profile for the Janus monolayer with and without gas adsorbed molecules is presented in Figure 6b,c for the Se/Te-side, respectively. Generally, the applied bias voltage influences the Fermi level of both left and right electrodes and tries to raise the Fermi level. It was seen that the left electrode conduction band minimum (CBM) approaches the right electrode and CBM indicates the variation in the current. Due to the metallic nature of the Janus NbSeTe monolayer, the linear increment of current is appeared with increasing of voltage with an interval of 0.1 V as shown in Figure 6b. The obtained I−V profile for the toxic gas molecules on both sides (i.e., Se/Te-side of the Janus monolayer) shows the different trends corresponding to the lower to a higher voltage (see Figure 6b,c). Overall, the current variation of the Janus NbSeTe monolayer with adsorbed molecules falls largely compared to the pure NbSeTe system, which is caused by the difference in the resistance offered by different types of molecules. The case of a pure Janus NbSeTe monolayer displayed the passing current of 64.5 μA at an applied bias voltage of 1.0 V. When gas molecules adsorbed on the surface of the Janus NbSeTe monolayer, the passing current suddenly reduced to 40.9, 27.0, 50.5, 35.2, 38.0, and 12.2 μA at the applied bias voltage 1.0 V for CO, CO${}_{2}$, NO, NO${}_{2}$, H${}_{2}$S, and SO${}_{2}$ gas molecules on the Se-side, respectively. Apart from this, we found the current flow of 60.1, 40.0, 13.8, 30.1, 24.34, and 24.30 μA through the Janus NbSeTe monolayer at applied bias voltage 1.0 V for CO, CO${}_{2}$, NO, NO${}_{2}$, H${}_{2}$S, and SO${}_{2}$ gas molecules on the Te-side, respectively. It can be noticed that the passing current for each molecule on the Te-side is significantly higher than that of the Se-side of the Janus NbSeTe monolayer which is strongly consistent with the charge transfer and binding energy for these adsorbed gas molecules except NO and NO${}_{2}$ gas. As a result of gas molecules adsorbed on Se-side, more resistance due to the larger interaction between the gas molecules and the Janus NbSeTe monolayer is created. In particular, the distinct current signal on both sides of the adsorbed gas molecules indicating with respect to different voltage shows better sensitivity and selectivity of Janus substrate towards gas molecules.

**Figure 8:**
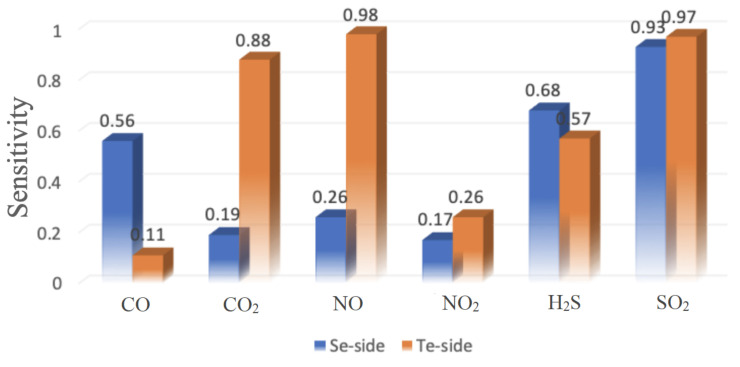
The sensitivity of CO, CO${}_{2}$, NO, NO${}_{2}$, H${}_{2}$S, and SO${}_{2}$ gas molecules on the surface of the Janus NbSeTe monolayer at a voltage of 0.3 V.

At last, the sensitivity of these toxic gases on the surface of the Janus NbSeTe monolayer is calculated as follows,

(6) $$S=\frac{|I-I_{0}|}{I_{0}}$$

where *I* and $I_{0}$ are current with and without adsorbed gas molecules on the surface of the Janus NbSeTe monolayer, respectively. The sensitivity of these gas molecules on both the Se/Te-side is presented in Table 2 on the surface of the Janus monolayer. It is confirmed that the Te-side is more sensitive as compare to the Se-side for the entire applied bias voltage. The maximum sensitivity is found to be 0.98 and 0.97 at 0.3 V and 0.1 V for NO and H${}_{2}$S gas molecules on the Te-side, respectively. Figure 8 shows the sensitivity for gas sensing devices of CO, CO${}_{2}$, NO, NO${}_{2}$, H${}_{2}$S, and SO${}_{2}$ gas molecules on both sides of the Janus NbSeTe monolayer at the voltage of 0.3 V. Our finding values of sensitivity of these gases are relatively good as compared to the previously reported work on 2D layered materials for different gas molecules [75,76,77]. These characteristics of the Janus monolayer provide great potential applications for sensing toxic gas in the environment.

## 4. Conclusions

In summary, the two-dimensional Janus NbSeTe monolayer is found to be a suitable candidate for gas detection devices for the detection of toxic gases present in the environment. We have systematically investigated the toxic gas (CO, CO${}_{2}$, NO, NO${}_{2}$, H${}_{2}$S, and SO${}_{2}$) sensing mechanism by adsorption energy, charge transfer, and electronic transport properties of the Janus NbSeTe monolayer using first-principles density functional theory methods. Our results suggest that the binding energies for these gases are below −1 eV and it shows physisorption interactions with Janus monolayer. We further confirm the adsorption behavior in terms of charge transfer analysis and significant variation in the work function for gas molecules situated on the surface of the Janus monolayer. From the charge transfer mechanism, it has been found that these gas molecules act as electron acceptors and the Janus NbSeTe monolayer act as an electron donor. In addition, a fast recovery time has been observed for each considered gas which is a very important finding as it determines the worthiness of a sensor material’s recyclability. It was seen that the extremely low recovery times for NO, NO${}_{2}$ gases in the order of μs and other gases such as CO, CO${}_{2}$, H${}_{2}$S, and SO${}_{2}$ are in the order of ps. With the view of a sensor device for electrical gas detection, we have calculated further transmission functions, I−V characteristics, and sensitivity of gas molecules with Janus monolayer. A detectable quenching of transmission spectra and I−V characteristics were observed as a result of the adsorption of these gases, which can be considered as OFF and ON states of the detection mechanism. Based on these findings, it is clear that the Janus monolayer can be a potentially important substrate for the detection of toxic gases and our study demonstrates the possibility of designing a high-performance nanosensor for toxic gas detection based on the Janus monolayer.

## Figures and Tables

**Table 1:** Calculated binding energy ($E_{b}$) when gas molecules absorbed on the surface of the Janus NbSeTe monolayer, charge transfer (▵Q) between gas molecules and Janus NbSeTe sheet, and work function (ϕ) variation during the gas molecules absorption process on the Se- and Te-side.

System	E${}_{b}($Se${}_{\mathrm{side}}$)	E${}_{b}($Te${}_{\mathrm{side}})$	$▵Q_{\mathrm{Se}}$	$▵Q_{\mathrm{Te}}$	$\phi_{\mathrm{Se}}$	$\phi_{\mathrm{Te}}$
CO	−0.15	−0.12	−0.0159	−0.0065	4.946	5.083
CO${}_{2}$	−0.20	−0.19	−0.0157	−0.0075	5.092	5.092
NO	−0.55	−0.53	−0.1297	0.024	4.99	5.044
NO${}_{2}$	−0.59	−0.68	−0.284	−0.533	5.253	5.098
H${}_{2}$S	−0.18	−0.13	−0.092	0.0235	5.157	5.267
SO${}_{2}$	−0.26	−0.25	−0.1614	−0.135	5.338	5.366

**Table 2:** Calculated work function (ϕ) when gas molecules absorbed on the surface of the Janus NbSeTe monolayer, recovery time (τ) at room temperature, and sensitivity (*S*) at a particular voltage.

System	$\phi_{\mathrm{Se}}$	$\phi_{\mathrm{Te}}$	$\tau_{\mathrm{Se}}$	$\tau_{\mathrm{Te}}$	$S_{\mathrm{Se}}$	$S_{\mathrm{Te}}$
CO	4.95	5.08	2.8 ps	1.4 ps	0.58 (0.2 V)	0.39 (0.1 V)
CO${}_{2}$	5.09	5.09	23.1 ps	13.3 ps	0.72 (0.1 V)	0.88 (0.3 V)
NO	4.99	5.04	17.49 μs	8.07 μs	0.34 (0.5 V)	0.98 (0.3 V)
NO${}_{2}$	5.25	5.10	75.74 μs	3015.54 μs	0.45 (1 V)	0.56 (0.9 V)
H${}_{2}$S	5.17	5.27	11.9 ps	1.78 ps	0.68 (0.3 V)	0.70 (0.2 V)
SO${}_{2}$	5.34	5.36	251.9 ps	204.6 ps	0.94 (0.5 V)	0.97 (0.3 V)

## References

[1] Billings P. (2007). Emission of hazardous air pollutants from coal-fired power plants. Environ. Health Eng..

[2] American Lung Association (2012). Toxic Air: The Case for Cleaning Up Coal-Fired Power Plants.

[3] Hong M.K., Bero L.A. (2002). How the tobacco industry responded to an influential study of the health effects of secondhand smoke. BMJ.

[4] Terrill J.B., Montgomery R.R., Reinhardt C.F. (1978). Toxic gases from fires. Science.

[5] Bessac B.F., Jordt S.E. (2010). Sensory detection and responses to toxic gases: Mechanisms, health effects, and countermeasures. Proc. Am. Thorac. Soc..

[6] Fisher A.B. (1980). Oxygen therapy: Cide effects and toxicity. Am. Rev. Respir. Dis..

[7] Assael M.J., Kakosimos K.E. (2010). Fires, Explosions, and Toxic Gas Dispersions: Effects Calculation and Risk Analysis.

[8] Late D.J., Doneux T., Bougouma M. (2014). Single-layer MoSe_2_ based NH_3_ gas sensor. Appl. Phys. Lett..

[9] Afzal A., Cioffi N., Sabbatini L., Torsi L. (2012). NO*_x_* sensors based on semiconducting metal oxide nanostructures: Progress and perspectives. Sens. Actuators Chem..

[10] Novoselov K., Jiang D., Schedin F.J., Booth T., Khotkevich V.V., Morozov S.V., Geim A.K. (2005). Two-dimensional atomic crystals. Proc. Natl. Acad. Sci. USA.

[11] Schedin F., Geim A.K., Morozov S.V., Hill E.W., Blake P., Katsnelson M.I., Novoselov K.S. (2007). Detection of individual gas molecules adsorbed on graphene. Nat. Mater..

[12] Kaewmaraya T., Ngamwongwan L., Moontragoon P., Jarernboon W., Singh D., Ahuja R., Karton A., Hussain T. (2020). Novel green phosphorene as a superior chemical gas sensing material. J. Hazard. Mater..

[13] Vogt P., De Padova P., Quaresima C., Avila J., Frantzeskakis E., Asensio M.C., Resta A., Ealet B., Le Lay G. (2012). Silicene: Compelling experimental evidence for graphenelike two-dimensional silicon. Phys. Rev. Lett..

[14] Gupta S.K., Singh D., Rajput K., Sonvane Y. (2016). Germanene: A new electronic gas sensing material. RSC Adv..

[15] Singh D., Gupta S.K., Sonvane Y., Lukačević I. (2016). Antimonene: A monolayer material for ultraviolet optical nanodevices. J. Mater. Chem. C.

[16] Singh D., Gupta S.K., Lukačević I., Sonvane Y. (2016). Indiene 2D monolayer: A new nanoelectronic material. RSC Adv..

[17] Singh D., Gupta S.K., Sonvane Y., Sahoo S. (2017). Modulating the electronic and optical properties of monolayer arsenene phases by organic molecular doping. Nanotechnology.

[18] Radisavljevic B., Radenovic A., Brivio J., Giacometti V., Kis A. (2011). Single Layer MoS_2_ Transistors. Nat. Nano.

[19] Pandey K., Yadav P., Singh D., Gupta S.K., Sonvane Y., Lukačević I., Kim J., Kumar M. (2016). First step to investigate nature of electronic states and transport in flower-like MoS_2_: Combining experimental studies with computational calculations. Sci. Rep..

[20] Singh D., Gupta S.K., Sonvane Y., Kumar A., Ahuja R. (2016). 2D-HfS_2_ as an efficient photocatalyst for water splitting. Catal. Sci. Technol..

[21] Singh D., Kansara S., Gupta S.K., Sonvane Y. (2018). Single layer of carbon phosphide as an efficient material for optoelectronic devices. J. Mater. Sci..

[22] Singh D., Gupta S.K., Sonvane Y., Ahuja R. (2017). High performance material for hydrogen storage: Graphenelike Si_2_BN solid. Int. J. Hydrogen Energy.

[23] Mishra P., Singh D., Sonvane Y., Ahuja R. (2020). Two-dimensional boron monochalcogenide monolayer for thermoelectric material. Sustain. Energy Fuels.

[24] Huang J., Chu J., Wang Z., Zhang J., Yang A., Li X., Gao C., Huang H., Wang X., Cheng Y. (2019). Chemisorption of NO_2_ to MoS_2_ Nanostructures and its Effects for MoS_2_ Sensors. ChemNanoMat.

[25] Zhang L., Khan K., Zou J., Zhang H., Li Y. (2019). Recent Advances in Emerging 2D Material-Based Gas Sensors: Potential in Disease Diagnosis. Adv. Mater. Interfaces.

[26] Singh D., Shukla V., Panda P.K., Mishra Y.K., Rubahn H.G., Ahuja R. (2020). Carbon-phosphide monolayer with high carrier mobility and perceptible I–V response for superior gas sensing. New J. Chem..

[27] Jiang S., Hu Y., Wu H., Zhang Y., Zhang Y., Wang Y., Zhang Y., Zhu W., Li J., Wu D. (2019). Multifunctional Janus Microplates Arrays Actuated by Magnetic Fields for Water/Light Switches and Bio-Inspired Assimilatory Coloration. Adv. Mater..

[28] Wang Y., Xiao J., Zhu H., Li Y., Alsaid Y., Fong K.Y., Zhou Y., Wang S., Shi W., Wang Y. (2017). Structural phase transition in monolayer MoTe_2_ driven by electrostatic doping. Nature.

[29] Gong C., Zhang X. (2019). Two-dimensional magnetic crystals and emergent heterostructure devices. Science.

[30] Yu X.-F., Li Y.-C., Cheng J.-B., Liu Z.-B., Li Q.-Z., Li W.-Z., Yang X., Xiao B. (2015). Monolayer Ti_2_CO_2_: A promising candidate for NH_3_ sensor or capturer with high sensitivity and selectivity. ACS Appl. Mater. Interfaces.

[31] Ai W., Kou L., Hu X., Wang Y., Krasheninnikov A.V., Sun L., Shen X. (2019). Enhanced sensitivity of MoSe_2_ monolayer for gas adsorption induced by electric field. J. Phys. Condens. Matter.

[32] Lu A.Y., Zhu H., Xiao J., Chuu C.P., Han Y., Chiu M.H., Cheng C.C., Yang C.W., Wei K.H., Yang Y. (2017). Janus monolayers of transition metal dichalcogenides. Nat. Nanotechnol..

[33] Zhang J., Jia S., Kholmanov I., Dong L., Er D., Chen W., Guo H., Jin Z., Shenoy V.B., Shi L. (2017). Janus monolayer transition-metal dichalcogenides. ACS Nano.

[34] Yang X., Singh D., Xu Z., Wang Z., Ahuja R. (2019). An emerging Janus MoSeTe material for potential applications in optoelectronic devices. J. Mater. Chem. C.

[35] Yang X., Singh D., Xu Z., Ahuja R. (2020). Sensing the polar molecules MH_3_ (M = N, P, or As) with a Janus NbTeSe monolayer. New J. Chem..

[36] Ma X., Wu X., Wang H., Wang Y. (2018). A Janus MoSSe monolayer: A potential wide solar-spectrum water-splitting photocatalyst with a low carrier recombination rate. J. Mater. Chem. A.

[37] Li F., Wei W., Zhao P., Huang B., Dai Y. (2017). Electronic and optical properties of pristine and vertical and lateral heterostructures of Janus MoSSe and WSSe. J. Phys. Chem. Lett..

[38] Meng M., Li T., Li S., Liu K. (2018). Ferromagnetism induced by point defect in Janus monolayer MoSSe regulated by strain engineering. J. Phys. Appl. Phys..

[39] Guo S.D. (2018). Phonon transport in Janus monolayer MoSSe: A first-principles study. Phys. Chem. Chem. Phys..

[40] Cai H., Guo Y., Gao H., Guo W. (2019). Tribo-piezoelectricity in Janus transition metal dichalcogenide bilayers: A first-principles study. Nano Energy.

[41] Long C., Gong Z.R., Jin H., Dai Y. (2018). Observation of intrinsic dark exciton in Janus-MoSSe heterosturcture induced by intrinsic electric field. J. Phys. Condens. Matter.

[42] Wang M., Pang Y., Liu D.Y., Zheng S.H., Song Q.L. (2018). Tuning magnetism by strain and external electric field in zigzag Janus MoSSe nanoribbons. Comput. Mater. Sci..

[43] Jin C., Tang X., Tan X., Smith S.C., Dai Y., Kou L. (2019). A Janus MoSSe monolayer: A superior and strain-sensitive gas sensing material. J. Mater. Chem. A.

[44] Wang D., Lan T., Pan J., Liu Z., Yang A., Yang M., Chu J., Yuan H., Wang X., Li Y. (2020). Janus MoSSe monolayer: A highly strain-sensitive gas sensing material to detect SF_6_ decompositions. Sens. Actuators A Phys..

[45] Wu Q., Cao L., Ang Y.S., Ang L.K. (2020). Superior and tunable gas sensing properties of Janus PtSSe monolayer. Nano Express.

[46] Chaurasiya R., Dixit A. (2019). Defect engineered MoSSe Janus monolayer as a promising two dimensional material for NO_2_ and NO gas sensing. Appl. Surf. Sci..

[47] Yang X.Y., Hussain T., Wärnå J.P.A., Xu Z., Ahuja R. (2021). Exploring Janus MoSSe monolayer as a workable media for SOF_6_ decompositions sensing based on DFT calculations. Comput. Mater. Sci..

[48] Chaurasiya R., Dixit A. (2020). Ultrahigh sensitivity with excellent recovery time for NH_3_ and NO_2_ in pristine and defect mediated Janus WSSe monolayer. Phys. Chem. Chem. Phys..

[49] Dou K.P., Hu H.H., Wang X., Wang X., Jin H., Zhang G.P., Shi X.Q., Kou L. (2020). Asymmetrically flexoelectric gating effect of Janus transition-metal dichalcogenides and their sensor applications. J. Mater. Chem. C.

[50] Kresse G., Joubert D. (1999). From ultrasoft pseudopotentials to the projector augmented-wave method. Phys. Rev. B.

[51] Kresse G., Furthmüller J. (1996). Efficiency of ab-initio total energy calculations for metals and semiconductors using a plane-wave basis set. Comput. Mater. Sci..

[52] Kresse G., Furthmüller J. (1996). Efficient iterative schemes for ab initio total-energy calculations using a plane-wave basis set. Phys. Rev. B.

[53] Perdew J.P., Burke K., Ernzerhof M. (1996). Generalized gradient approximation made simple. Phys. Rev. Lett..

[54] Dion M., Rydberg H., Schröder E., Langreth D.C., Lundqvist B.I. (2004). Van der Waals density functional for general geometries. Phys. Rev. Lett..

[55] Monkhorst H.J., Pack J.D. (1976). Special points for Brillouin-zone integrations. Phys. Rev. B.

[56] Blöchl P.E. (1994). Projector augmented-wave method. Phys. Rev. B.

[57] Ruiz-Serrano Á., Hine N.D., Skylaris C.K. (2012). Pulay forces from localized orbitals optimized in situ using a psinc basis set. J. Chem. Phys..

[58] Shen X., Sun L., Benassi E., Shen Z., Zhao X., Sanvito S., Hou S. (2010). Spin filter effect of manganese phthalocyanine contacted with single-walled carbon nanotube electrodes. J. Chem. Phys..

[59] Rocha A.R., García-Suárez V.M., Bailey S., Lambert C., Ferrer J., Sanvito S. (2006). Spin and molecular electronics in atomically generated orbital landscapes. Phys. Rev. B.

[60] Román-Pérez G., Soler J.M. (2009). Efficient implementation of a van der Waals density functional: Application to double-wall carbon nanotubes. Phys. Rev. Lett..

[61] Datta S. (1997). Electronic Transport in Mesoscopic Systems.

[62] Yang X., Banerjee A., Ahuja R. (2020). Structural Insight of the Frailty of 2D Janus NbSeTe as an Active Photocatalyst. ChemCatChem.

[63] Ding Y., Wang Y., Ni J., Shi L., Shi S., Tang W. (2011). First principles study of structural, vibrational and electronic properties of graphene-like MX_2_ (M = Mo, Nb, W, Ta; X = S, Se, Te) monolayers. Phys. B Condens. Matter.

[64] Hussain T., Singh D., Gupta S.K., Karton A., Sonvane Y., Ahuja R. (2019). Efficient and selective sensing of nitrogen-containing gases by Si_2_BN nanosheets under pristine and pre-oxidized conditions. Appl. Surf. Sci..

[65] Leenaerts O., Partoens B., Peeters F. (2008). Adsorption of H_2_O, NH_3_, CO, NO_2_, and NO on graphene: A first-principles study. Phys. Rev. B.

[66] Liu Y., Wilcox J. (2011). CO_2_ adsorption on carbon models of organic constituents of gas shale and coal. Environ. Sci. Technol..

[67] Feng J., Liu Y., Wang H., Zhao J., Cai Q., Wang X. (2014). Gas adsorption on silicene: A theoretical study. Comput. Mater. Sci..

[68] Xia W., Hu W., Li Z., Yang J. (2014). A first-principles study of gas adsorption on germanene. Phys. Chem. Chem. Phys..

[69] Kou L., Frauenheim T., Chen C. (2014). Phosphorene as a superior gas sensor: Selective adsorption and distinct I–V response. J. Phys. Chem. Lett..

[70] Shokri A., Salami N. (2016). Gas sensor based on MoS_2_ monolayer. Sens. Actuators B Chem..

[71] Cho B., Hahm M.G., Choi M., Yoon J., Kim A.R., Lee Y.J., Park S.G., Kwon J.D., Kim C.S., Song M. (2015). Charge-transfer-based gas sensing using atomic-layer MoS_2_. Sci. Rep..

[72] Singh D., Panda P.K., Mishra Y.K., Ahuja R. (2020). Van der Waals induced molecular recognition of canonical DNA nucleobases on a 2D GaS monolayer. Phys. Chem. Chem. Phys..

[73] Bano A., Krishna J., Pandey D.K., Gaur N. (2019). An ab initio study of sensing applications of MoB_2_ monolayer: A potential gas sensor. Phys. Chem. Chem. Phys..

[74] Kumar V., Azhikodan D., Roy D.R. (2020). 2D Sb_2_C_3_ monolayer: A promising material for recyclable gas sensor for environmentally toxic nitrogen-containing gases (NCGs). J. Hazard. Mater..

[75] Kumar V., Rajput K., Roy D.R. (2020). Monolayer Bi_2_C_3_: A promising sensor for environmentally toxic NCGs with high sensitivity and selectivity. Appl. Surf. Sci..

[76] Guo H., Zhang W., Lu N., Zhuo Z., Zeng X.C., Wu X., Yang J. (2015). CO_2_ capture on h-BN sheet with high selectivity controlled by external electric field. J. Phys. Chem. C.

[77] Rajput K., Kumar V., Roy D.R. (2020). Heterobilayer CaS/CaSe: A promising sensor for environmental toxic NO_2_ gas with high selectivity and sensitivity. Appl. Surf. Sci..

